# Effect of Bletilla Striata Polysaccharide on the Pasting, Rheological and Adhesive Properties of Wheat Starch

**DOI:** 10.3390/polym15244721

**Published:** 2023-12-15

**Authors:** Haibo Zhao, Qiang Xu, Tianlan Yan, Hongdong Zhang, Yuliang Yang

**Affiliations:** 1 Institute for Preservation and Conservation of Chinese Ancient Books, Fudan University Library, Fudan University, Shanghai 200433, China; 20110820004@fudan.edu.cn (H.Z.); xuqiang1209@163.com (Q.X.); 2 Department of Chemistry, Fudan University, Shanghai 200433, China; 19110220099@fudan.edu.cn; 3 State Key Laboratory of Molecular Engineering of Polymers, Department of Macromolecular Science, Fudan University, Shanghai 200433, China

**Keywords:** bletilla striata polysaccharide, modification, wheat starch, adhesive

## Abstract

A combination of starch and hydrocolloids is a facile method for physically modifying native starch. Bletilla striata polysaccharide (BSP) is a glucomannan with various applications in the food and cosmetic industries as a thickening agent. This study focused on investigating the impact of BSP on the pasting, rheological and adhesive properties of wheat starch (WS). Results from a Rapid Visco-Analyzer (RVA) revealed that the addition of BSP (below 0.2%) resulted in increases in peak viscosity, breakdown and setback values. However, for the addition of BSP at a higher concentration (0.3%), the opposite trend was observed. Rheological measurements indicated that the presence of BSP increased the viscoelastic properties of WS–BSP gels. TGA results demonstrated that the presence of BSP promoted the thermal stability of starch. FTIR results indicated the short-range order structure decreased at low addition concentrations of BSP (0.05% and 0.1%) and increased with higher BSP addition concentrations (0.2% and 0.3%). SEM observation showed that the BSP improved the hydrophilic property of starch gels and decreased the size of pores in the starch gels. Further, the mechanical properties of paper samples unveiled that the present of BSP in starch gels obviously increased its bonding strength as an adhesive.

## 1. Introduction

Starch, as a photosynthetic product, serves as the primary storage material in various plants like potato, wheat, and pea. In addition to its essential role in nutrition, starch has found broad applications in modern society [1]. One of the prominent uses of starch is as an adhesive in paper industry [2,3]. Additionally, starch is utilized in the production of biodegradable plastics and serves as a scaffold in tissue engineering applications [4,5]. Furthermore, starch can form a viscoelastic system through a pasting process [6]. This property has made it a favored adhesive in the restoration of paintings and calligraphy for centuries [7]. However, native starch has limitations, such as low viscosity and ease of retrogradation, which hinder its application as an adhesive [8,9]. Restorers, in practice, have found that natural starch gel is ineffective in bonding large-scale paper works. Consequently, physical modification methods through blending wheat starch with Bletilla striata extraction solution to alter the physicochemical properties of starch gel has been put into practice for more than hundreds of years in the field of mounting calligraphy and paintings [10]. The obtained starch gels can perfectly meet the specific requirements of restoration projects.

*Bletilla striata (Thumb.) Reichb. f.* is an orchidaceous herb that is widely cultivated in East Asia. It is traditionally used in Chinese medicine for its anti-inflammatory and hemostatic properties [11,12]. Prior investigations have indicated that the tuber of Bletilla striata contains a significant amount of water-soluble polysaccharide. BSP primarily consists of 1,4-linked mannosyl residues and 1,4-linked glucosyl residues [13]. Previous reports have highlighted the various applications of BSP in the food and cosmetic industries as a thickening agent [14]. Furthermore, BSP has gained attention for its potential use in developing novel biomaterials, specifically as wound dressings and drug vehicles [15]. Many researchers have incorporated different types of natural polysaccharides into starch mixtures to modify their pasting and retrogradation behaviors. For instance, Ren et al. demonstrated that Mesona chinensis Benth polysaccharide enhances the viscoelasticity of SPS-MCP gels by forming hydrogen bonds and electrostatic forces between SPS and MCP [16]. Kong et al. showed that Cordyceps polysaccharide can reduce the degree of starch gelatinization and inhibit short-term retrogradation of starch mixtures [17]. However, there is currently no research focused on the influence of BSP on the starch gelatinization.

The primary aim of this study is to examine the impact of various levels of BSP content on the pasting and rheological properties of WS. Moreover, another focus of this study is to examine the impact of BSP on the adhesive strength of WS–BSP gels. This investigation is significant as it would provide valuable insights into the scientific nature of conventional techniques for producing adhesives with a high bonding strength from starch by blending it with natural plant extracts. 

## 2. Materials and Methods

### 2.1. Materials

Wheat starch (amylose: 24.3%, water: 9.6%) was supplied by Shanghai Yuanye Bio-Technology Co., Ltd., Shanghai, China. The tubers of Bletilla striata were purchased from Anhui Guben Qiancao Biotechnology Co., Ltd., Hefei, China. They were crushed into powders by a microniser and then selected through a 60-mesh sieve. The BSP was extracted from the tuber following the method described by Chen et al. [18]. The BSP contained 98.2% total sugars with an average molecular weight of 1.42 × 10^5^ Da.

### 2.2. Sample Preparation

To prepare the WS (4%, *w*/*v*)–BSP (0, 0.05, 0.1, 0.2 and 0.3%, *w*/*v*) suspensions, the following procedure was followed. Initially, a specific amount of BSP was dissolved in water with one hour stirring at 60 °C to ensure complete dissolution. After the BSP solution was stabilized to 25 °C, WS was gradually introduced into the solution under consistent agitation, resulting in the formation of the desired mixtures.

### 2.3. Pasting Properties

The pasting properties of WS influenced by BSP were assessed using a RVA (TecMaster, Perten, Australia). To initiate the process, the BSP was dispersed in 25 mL distilled water under magnetic stirring. Subsequently, 2 g of starch was gradually introduced into the BSP solution. The samples were subjected to the thermal program (STD1). Initially, the slurries were maintained at 50 °C for 1.5 min, and then heated to 95 °C in 3.5 min. Subsequently, they were maintained at 95 °C for 2.5 min. Following the heating phase, the samples were gradually cooled to 50 °C in 3.5 min. They were then kept at 50 °C for an additional 4 min. During the analysis, the speed of the apparatus was set to 960 rpm for the first 10 s to ensure uniform dispersion, after which, it was adjusted to a constant speed of 160 rpm for the remainder of the testing period. During this process, the RVA instrument automatically detected and recorded the changes in viscosity of the sample over time. Peak viscosity (PV), trough viscosity (TV), breakdown, final viscosity (FV) and setback were determined from the RVA curves. 

### 2.4. Leaching Amylose and Swelling Power

The samples were prepared as detailed in Section 2.2. The slurries were then subjected to constant stirring and heated to 75 °C, 85 °C and 95 °C for 20 min. After the pasting process, the samples were cooled to 25 °C and then centrifuged at 4800 rpm for 30 min. The leaching amylose in the mixtures was obtained according to the method reported by Chrastil [19]. The swelling power of the mixtures was calculated according to Equation (1) [20].

Swelling power (%) = B/A × 100% (1)

A is the sediment weight after centrifugation, g; B is the dry basis of sediment, g.

### 2.5. DSC

The thermal analysis of WS–BSP mixtures was conducted by DSC 250 (TA Instruments, New Castle, DE, USA) [21]. In detail, WS (3 mg) was mixed with 6 μL BSP solution (0, 0.05, 0.1, 0.2 and 0.3%, *w*/*v*) and then enclosed in an aluminum pan. This procedure ensured the uniform dispersion of starch granules within the continuous phase, while allowing for the uniform envelopment of BSP on the granule surfaces. The samples should be equilibrated at 25 °C for 12 h to ensure full hydration of granules, and then, the aluminum pans were transferred to the instrument and then heated from 35 °C and 95 °C at a rate of 10 °C/min. Special software (TRIOS software version 5.4.0.300) was used to evaluate the gelatinization parameters, including onset temperature (To), peak temperature (Tp), concluding temperature (Tc) and the area of the main endothermic peak (J/g). 

### 2.6. Rheological Measurements

The WS–BSP gels, prepared according to the description in Section 2.4 at 95 °C, were stored at room temperature for 24 h for further testing. The rheological results were obtained by a rheometer at 25 °C (HAAKE MARS III, Thermofisher, Waltham, MA, USA). A parallel plate geometry was employed (35 mm diameters, 0.5 mm gap).

#### 2.6.1. Dynamic Rheological Properties

The WS–BSP gels were put on the rheometer plate and allowed to equilibrate for 5 min before the start of testing. A fixed strain of 1% was chosen for the subsequent frequency sweep experiment. This fixed strain ensures that the gel remains within the linear viscoelastic region [20]. The frequency range was set at 0.1–10 Hz for the frequency sweep experiment. The analysis of the frequency-dependent viscoelastic properties of the gel, both the storage modulus (G′) and the loss modulus (G″), was recorded [22].

#### 2.6.2. Steady Rheological Properties

In the steady shear experiments, a cyclic period of shear rates was employed to investigate the changes in apparent viscosity and shear stress of the mixtures against the shear rates (0.1 s^−1^ to 1000 s^−1^). The Herchel–Bulkley model as shown in Equation (2) was employed to analyze the results of the steady shear tests [23]:

τ = τ_0_ + Kx^n^ (2)

where τ is the shear stress (Pa); τ_0_ is the yield stress (Pa); K is the consistency coefficient (Pa·sn); γ is the shear rate (s^−1^); and n is the flow behavior coefficient.

### 2.7. ATR-FTIR

The preparation of WS–BSP gels was conducted as described in Section 2.6, and they were subsequently stored at −80 °C for 6 h. After freezing, the gels were subjected to a vacuum freeze dryer and the obtained dried samples were transferred to an ATR-FTIR instrument (Nicolet 6700, Thermofisher, Waltham, MA, USA) according to the method reported by Guo et al. with slight modification [24]. The spectral collection range was set from 4000 cm^−1^ to 500 cm^−1^, and each sample was measured at a resolution of 4 cm^−1^, with an accumulation of 64 scans.

### 2.8. TGA

The sample preparation was conducted as described in Section 2.7. Thermal gravimetric analysis (TGA, PE Pyris 1, Perkinelmer, Waltham, MA, USA) of the WS-BSP gels was carried out during the temperature range of 30–600 °C under a N_2_ atmosphere, with a heating rate set at 20 °C/min [25,26]. The variation in weight loss proportion was recorded as the temperature increased.

### 2.9. SEM

The structure of the samples, prepared as described in Section 2.7, was observed using an SEM ((Zeiss, Ultra 55, Oberkochen, Germany). The samples were transferred to the loading platform by conductive adhesive tape then coated with gold. The accelerating voltage of the SEM was set at 20 kV [27].

### 2.10. Paper Mechanical Properties

The WS–BSP gels were prepared according to the procedures outlined in Section 2.6. The obtained WS–BSP gels were coated uniformly onto a single sheet of paper measuring 0.25 m × 0.25 m. The coating process was carried out using a coir scrub brush, ensuring that the gel was spread uniformly across the paper surface. After coating, another piece of paper was placed on top of the gel-coated paper and pressed together, adhering the two pieces of paper with the gel layer in between. This step is performed to create a sandwich-like structure with the gel layer securely enclosed between two paper layers. Once the gel-coated paper samples were assembled, they were allowed to dry at 30 °C for 24 h. The obtained dried paper samples with the gel layer were cut into different sizes for further testing [28]. The tensile strength, folding endurance and tearing strength experiments were carried out according to the methods reported by Zhao et al. [29].

### 2.11. Statistical Analysis

The significance of collected data was evaluated through one-way analysis of variance (ANOVA) using SPSS 20.0 (SPSS Inc., Chicago, IL, USA), with a significance level of *p* < 0.05.

## 3. Results and Discussion

### 3.1. Pasting Properties

The pasting characteristics of WS–BSP gels with varying BSP ratios were examined using an RVA instrument and the corresponding pasting parameters are displayed in Table 1. The pasting of starch is an intricate procedure encompassing a multitude of alterations in starch granules, encompassing the absorption of water, expansion, disruption of crystal structures and the release of amylose and partial amylopectin. Peak viscosity (PV) refers to the maximum viscosity attained by the starch mixture before the onset of cooling. In this study, with the addition concentration of BSP at 0.2%, the PV value of the WS–BSP mixture exhibits a maximum value of 1181 cp. BSP as a hydrophilic glucomannan has the capability to envelop the surface of starch particles, thereby impeding the absorption of water and the expansion of the granules, as well as the dissolution of soluble starch [30]. Simultaneously, the adsorption concentrations of BSP on the surface of starch granules also escalated, augmenting the resistance of starch granules to intermolecular movement. This subsequently led to an elevation in peak viscosity during the pasting procedure [31]. However, with a higher addition concentration of BSP (0.3%), there is a reduction in the PV value to 1004 cp. This phenomenon could be ascribed to the intensified concentration of BSP in the continuous phase, which led to subsequent phase separation [32] and prompted the aggregation of starch granules or ghosts within the dispersed phase. Consequently, this aggregation decreased the resistance of starch particles to intermolecular movement and resulted in a reduction in peak viscosity. The trough viscosity (TV) value refers to the lowest viscosity of mixtures after the breakage of swelling granules. The change in TV values with the addition of BSP is consistent with the trend of PV values. However, the difference in TV values with the addition of BSP was not obvious, which was due to the higher percentage of breakage among the swelling granules in the group with higher PV values. The breakdown viscosity (BD) value serves as an indicator of shear resistance and thermal stability of swollen granules. A higher BD value signifies that the starch pasting process is more susceptible to impairment. As the BSP concentration in the continuous phase rises, the escalating phase separation exerts greater external force on the granule surface, thereby facilitating the rupture of the starch granule. However, with a subsequent increase in the BSP concentration (0.3%) in the continuous phase, a more substantial two-phase separation occurred, resulting in the clustering of starch granules. Consequently, the shearing effect of external forces on the starch granule was diminished. Therefore, as the BSP concentration in the mixtures increased, the degree of starch granule breakage initially increased but subsequently decreased. The final viscosity (FV) refers to the viscosity of the resulting mixtures after the pasting process and mixtures with higher FV values indicate increased entanglement between the BSP and starch or a greater aggregation of soluble starch in the continuous phase. The setback viscosity (SB) refers to the short-term retrogradation of leaching amylose. The SB value also exhibits a maximum at a BSP addition concentration of 0.2%. The increased degree of starch breakage with increasing concentrations of BSP would lead to more amylose leaching out; although, the amount of leached amylose could not be detected due to the enveloping effect of BSP on the surface of starch ghosts, as suggested by the leaching amylose measurements in Section 3.2. However, as the concentration of BSP in the continuous phase and the levels of starch ghosts and leaching amylose in the dispersion phase increased, there was a tendency for phase separation to occur. This facilitated an enhanced interaction among starch molecules, consequently promoting the occurrence of short-term retrogradation. Notably, an increase in the BSP concentration led to a more pronounced degree of phase separation within the system. However, when BSP was added to a higher concentration (0.3%), the breakage of starch particles is inhibited and less soluble starch is released, resulting in fewer starch molecules participating in the short-term retrogradation process, consequently reducing the SB value to 576 cp.

### 3.2. Leaching Amylose and Swelling Power

**Figure 1:**
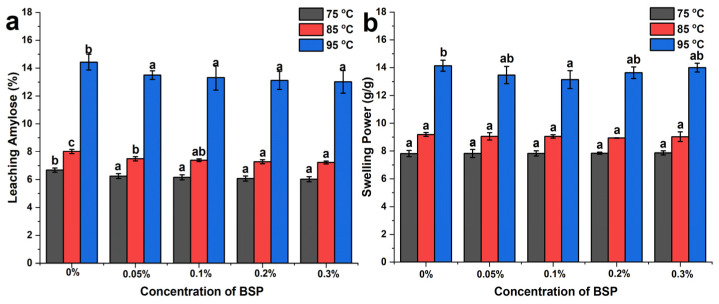
(**a**) The leaching amylose and (**b**) swelling power of WS–BSP gels at different temperature. The results were presented as mean ± standard deviations within the same column for each sample, with the application of different lowercases to indicate significant differences (*p* < 0.05).

The leaching amylose refers to the breakage of granules during the pasting process and the swelling power of the WS–BSP mixtures refers to the water holding capability of starch granules. The leaching amylose and swelling power of WS–BSP mixtures at different temperature are displayed in Figure 1. As shown in Figure 1, the increasing temperature can promote the leaching out of amylose and enhance the swelling power of starch granules. When the WS–BSP mixtures were subjected to heating at 75 °C, a temperature lower than the full pasting temperature, the starch granules retained their integrity, with only a few larger swollen granules being susceptible to external stress. The amount of amylose decreased with the addition of BSP, and the swelling power exhibited no significant difference. Upon reaching a temperature of 85 °C, the starch granules exhibited increased water absorption and swelling. Consequently, a greater amount of amylose could leach out from the granules when subjected to stirring. When at the pasting temperature of 95 °C, the increasing addition concentration of BSP in the mixtures led to a decrease in the amount of leaching amylose, which can be related to two reasons as mentioned in Section 2.1. Firstly, the presence of BSP reduces the water activity, limiting the water available for starch granules and reducing the swelling power of starch granules [33]. Secondly, the physical encasement of BSP on the granule surface hinders the release of amylose [34]. However, the swelling power of WS–BSP mixtures exhibits different phenomenon as depicted in Figure 1b. The presence of BSP at low addition concentrations (0.05% and 0.1%) results in a reduction in the water absorbing capacity of starch granules, which can be attributed to the stability of starch granules covered by BSP on its surface, leading to lower swelling power. With an increasing concentration of BSP (0.2% and 0.3%), the swelling power of the starch granules increases. This result can be attributed to the hydrophilic property of BSP, which envelops the surface of starch granules [35]. Consequently, the swelling power of granules is enhanced.

### 3.3. Thermal Properties

The gelatinization parameters derived from the DSC curves are shown in Table 2. The onset, peak and concluding temperature are not notably influenced by the presence of BSP, which is consistent with the results reported by Banchathanakij et al. [36]. The reason can be attributed to the fact that the increasing concentration of BSP in the continuous phase does not impact the thermal transition behavior of the solution. Meanwhile, the enthalpy of gelation (ΔH) of the samples showed no significant reduction with the addition of BSP. This phenomenon can be explained by the enveloping effect of BSP on the surface of starch granules, which hinders the water adsorption of starch granules and then renders the disintegration of the starch granules, further leading to no significant reduction in the values of gelatinization enthalpy. This finding is consistent with the results observed in the determination of leaching amylose.

### 3.4. Rheological Measurements

#### 3.4.1. Dynamic Rheological Properties

**Figure 2:**
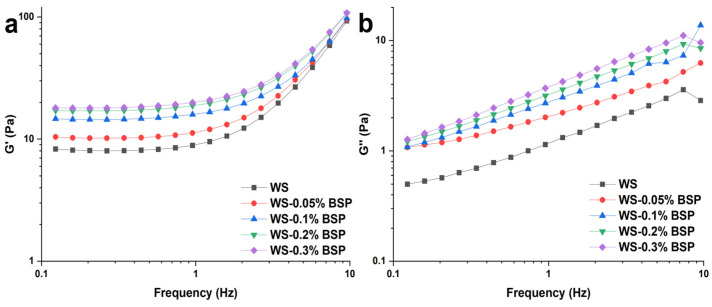
The results of dynamic rheological measurements of WS–BSP gels. (**a**) G′ and (**b**) G″ as a function of frequency at 1% strain.

The dynamic rheological measurement results for WS–BSP systems after 24 h of room temperature equilibration are depicted in Figure 2. The increase in G′ and G″ with the increasing frequency indicates that the WS–BSP mixtures display frequency dependence, particularly after 1 Hz. The G′ and G″ of mixtures represent the elastic characteristic and the viscous characteristic, respectively. As the addition of BSP to the systems increases, the G″ of WS-BSP increases notably. This phenomenon can be attributed to the increasing concentration of BSP in the continuous phase and then more BSP wraps the surface of starch ghosts, which contributes to the increased viscous characteristics of the systems [37]. Nonetheless, at higher BSP concentrations (0.3%), the rise in the G′ is less conspicuous when contrasted with the increased amplitude observed at lower BSP addition concentrations (0.05, 0.1 and 0.2%). The increasing BSP concentrations facilitate the breakage of starch granules which is consistent with the results of BD. BSP also augments the interactions among the starch molecules leached from the starch granules or the starch ghosts in the dispersion phase. However, at higher BSP addition concentrations, fewer soluble starch molecules engage in the formation of the gel network, leading to a marginal increase in the storage modulus [38].

#### 3.4.2. Steady Rheological Properties

**Figure 3:**
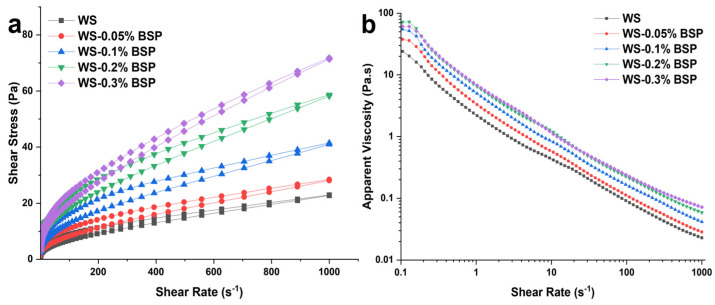
The results of steady rheological measurements conducted on WS–BSP gels. (**a**) Shear stress and (**b**) apparent viscosity, each illustrated as a function of shear rate.

The steady rheological measurement results for WS–BSP systems after 24 h of room temperature equilibration are depicted in Figure 3, and the corresponding steady rheological parameters fitted with the Herschel–Bulkley model are shown in Table 3. The R-squared values for all samples exceed 0.995, which signifies a good model fit. The findings of rheological measurements indicate the ability of WS–BSP systems to withstand external forces, with higher apparent viscosity or shear stress in WS–BSP gels signifying a greater entanglement between macromolecules. The WS–BSP gels have a typical pseudoplastic fluid behavior with shear-thinning properties, as indicated by an n value (0.44–0.57) notably lower than 1 [17]. The τ_0_ represents the initial stress applied to the WS–BSP gels, which is related to the movement of starch fragments past each other. As indicated in Table 3, the addition of BSP necessitates more initial stress to alter the initial shape of the WS–BSP gels. The consistency index (K) denotes the viscosity of the gels. Table 3 reveals that the K value reaches a peak at a BSP concentration of 0.2% compared to the control group. However, with higher BSP addition (0.3%), the K value decreases. This trend corresponds with the RVA measurement findings, indicating that the FV within the system reaches its maximum at a BSP addition concentration of 0.2%. The thixotropy of WS–BSP gels refers to the capacity of starch gels to return to their original structure, which can be assessed by observing the size of the hysteresis loop. A larger area of the hysteresis loop implies a more condensed gel structure. As depicted in Table 3, the WS–BSP gels with the addition of BSP at 0.2% exhibit the most condensed gel structure, aligning with the findings of the short-term retrogradation rate by the RVA measurements.

### 3.5. ATR-FTIR

**Figure 4:**
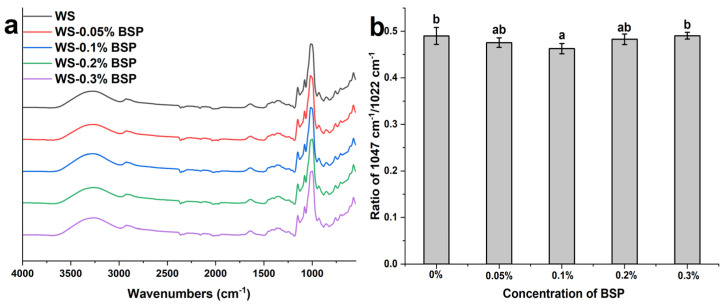
(**a**) The results of FT-IR analysis. (**b**) The ratio of adsorption at 1047 cm^−1^ to that at 1022 cm^−1^. The results are presented as mean ± standard deviations within the same column for each sample, with the application of different lowercases to indicate significant differences (*p* < 0.05).

Figure 4a presents the FT-IR spectrum of WS–BSP gels at different addition concentrations of BSP. There are no new adsorption peaks in the spectrum of the WS–BSP gels in contrast to the blank sample, signifying no new covalent bonding formation in the WS–BSP gels [39]. The absorption peaks in the range of 3100–3700 cm^−1^ refer to the bending vibration of O-H bonds within the starch molecule or BSP. And absorption peaks in the 1550–1750 cm^−1^ refer to the bonding water in the samples [40]. The spectral region between 1200 cm^−1^ and 800 cm^−1^ corresponds to the short-range ordered structure of samples. The values obtained from the spectra after deconvolution at 1022 cm^−1^ and 1047 cm^−1^ represent the disordered and ordered structures, respectively. As depicted in Figure 4b, the ratio of absorbance at 1047 cm^−1^ to 1022 cm^−1^ reflects the short-range ordered structure of WS–BSP gels [41]. At lower BSP addition concentrations (0.05% and 0.1%), the proportion of the short-range ordered structure decreases with an increasing concentration of BSP. Conversely, with higher BSP additions (0.2% and 0.3%), there is an increase in the proportion of the short-range ordered structure. As shown in the RVA test results, the low concentration of BSP solution increases the breakage degree of starch granules, but the amount of leaching amylose decreases due to the increase in BSP concentration, as indicated by the leaching amylose experiment. And at the same time, the degree of phase separation is not serious, resulting in less starch molecules to interact with each other, hence the short-range ordered structure decreases. However, although the amount of leaching amylose reduces further at high concentrations of BSP (0.2% and 0.3%), the phase separation is also increased, leading to the further aggregation of starch molecules, whether in the continuous phase or the dispersed phase, ultimately enhancing the short-range ordered structure.

### 3.6. TGA

**Figure 5:**
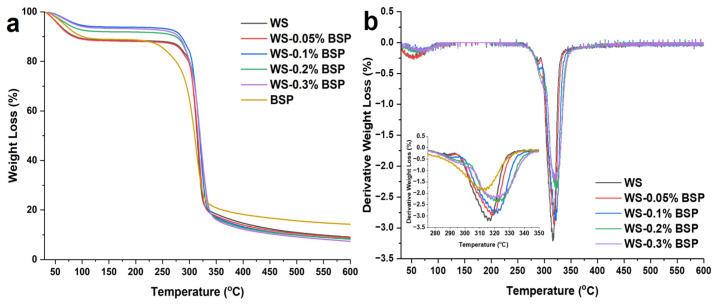
(**a**) The TGA curves of WS–BSP gels. (**b**) The first derivative of TGA results.

The TGA results are presented in Figure 5a, and the first derivative results are displayed in Figure 5b. There are primarily three decomposition stages in WS–BSP gels. The first decomposition stage from 30 °C to 110 °C corresponds to the loss of bound water in the starch gels [42]. The second decomposition stage occurs between 250 °C and 350 °C, which involves the cleavage of glycosidic bonds and is the principal phase of degradation in WS–BSP gels [43]. Remarkably, the addition of BSP shifts the degradation temperature to a higher value, and this can be attributed to the increase in BSP concentrations facilitating the degree of phase separation, thus increasing the interaction between starch fragments and improving the thermal stability of starch gels. The third decomposition stage commences at 350 °C and extends to 600 °C. During this phase, further glycosidic bond degradation and the breakdown of polymer fragments occur [26].

### 3.7. SEM 

**Figure 6:**
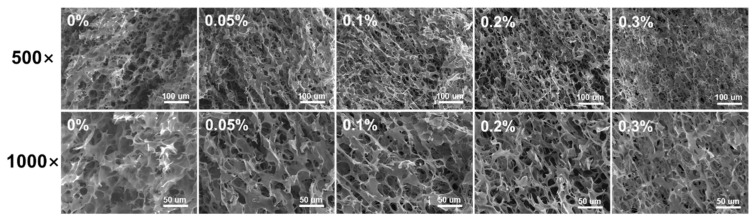
The SEM images of WS–BSP gels (500× and 1000× magnifications).

The SEM images of WS–BSP gels are depicted in Figure 6, with two levels of magnification. The WS-BSP gels exhibit a characteristic honeycomb-like structure. In comparison to the control sample, as the BSP addition increases, the pore size of starch gels gradually decreases. Additionally, the incorporation of BSP results in a denser structure within the starch gel. The formation of pores in starch gels occurs during the freezing process, where starch molecules interact with each other, thus causing the extrusion of water molecules from the gel matrix [44]. The presence of BSP in the continuous phase can protect the starch fragments from further fragmentation. Simultaneously, the hydrophilic characteristics of BSP, whether within the continuous phase or enveloping the surface of starch ghost, improve the water holding capacity of WS–BSP mixtures, consequently reducing the proportion of free water. This results in a gradual reduction in the final gel pore size as the BSP concentration increases. 

### 3.8. Mechanical Properties

The utilization of WS–BSP gels for adhesive testing on paper and the subsequent multidimensional mechanical property analysis of the paper samples are summarized in Table 4. It is evident that the addition of BSP (0.3%) significantly enhances the mechanical properties compared to the control group. In detail, the tearing strength of paper increases from 3.47 mN·m^2^/s to 4.75 mN·m^2^/s, and the fold endurance increases from 1.89 to 2.47, while the tensile strength increases from 1.17 (kN/m) to 1.43 (kN/m). This underlines the fact that incorporating BSP into the starch gels can significantly augment its bonding strength to the paper. Moreover, the elongation of paper samples increases from 0.92% to 1.17%. This suggests that the presence of BSP in the starch gels can also enhance the flexibility of paper samples, which is a crucial consideration for traditional art and calligraphy mounting. Chinese traditional painting and calligraphy works, especially scrolls, often necessitate repeated unfurling. Paper with an excessive rigidity may engender the emergence of cracks in the works during the process of perusal. When using a starch gel as a paper adhesive, its effectiveness is influenced by two factors. One is the strength of the WS–BSP gel itself as the binding layer and the other is the permeability of the adhesive into the porous paper structure. Rheological test results indicate that with the incorporation of BSP, the G′ of WS–BSP gels gradually increases, signifying a progressive reinforcement of the starch gels as the binding layer. Simultaneously, with the increasing BSP content, the G″ of WS–BSP gels also increases, implying improved flowability and enhanced penetration of the starch fragments into the paper, consequently augmenting the adhesive strength of starch gels. Additionally, as a natural neutral polysaccharide, BSP maintains a nearly neutral pH, with no deleterious effects on the pH of starch gels. Traditional conservators frequently introduce potassium alum into starch gels to bolster their adhesive efficacy. However, potassium alum is susceptible to hydrolysis, resulting in the release of a substantial quantity of hydrogen ions. This action leads to a reduction in the pH of starch gels and exacerbates paper degradation, causing alterations in paper color, typically shifting towards deeper tones of yellow and red [29]. In contrast, the incorporation of BSP mitigates these concerns, preserving the inherent pH of the adhesive while concurrently enhancing its adhesion to the paper.

## 4. Conclusions

The study of BSP on the pasting, rheological and adhesive properties of wheat starch carries significant implications for the preservation of traditional art and calligraphy mounting techniques. The presence of BSP influences the peak viscosity of the paste, augments the integrity of starch granules and reduces the precipitation of soluble starch and gelatinization enthalpy. Rheological tests indicate that the WS–BSP system forms a weak gel, exhibiting shear-thinning behavior. The inclusion of BSP effectively increases the apparent viscosity and G″ of the starch gel. TGA results reveal an improvement in the thermal stability of starch gels in the presence of BSP. Furthermore, FTIR demonstrates that low concentrations of BSP reduce the short-range ordered structure of starch, while high concentrations of BSP have the opposite trend. SEM confirms that BSP effectively reduces the pore size of the starch gel and enhances the interconnectivity strength between the starch gel network structures. Paper mechanical tests demonstrate that the presence of BSP significantly enhances the bonding strength of the starch gel to paper. Incorporating BSP into the starch suspension represents a straightforward and effective method for modifying the physicochemical properties of natural wheat starch.

## Figures and Tables

**Table 1:** Pasting properties of WS with and without the addition of BSP.

BSP Conc.	PV (cP)	TV (cP)	BD (cP)	FV (cP)	SB (cP)
0%	899 ± 18 a	720 ± 14 a	179 ± 4 a	1093 ± 21 a	373 ± 7 a
0.05%	921 ± 20 a	737 ± 15 ab	184 ± 5 a	1132 ± 20 ab	395 ± 5 a
0.1%	955 ± 27 ab	740 ± 17 ab	215 ± 10 b	1180 ± 23 b	440 ± 6 b
0.2%	1181 ± 49 c	750 ± 20 b	431 ± 29 d	1448 ± 36 d	698 ± 16 d
0.3%	1004 ± 30 b	732 ± 14 ab	272 ± 16 c	1308 ± 28 c	576 ± 14 c

PV: pasting viscosity; TV: trough viscosity; BV: breakdown viscosity; FV: final viscosity; SB: setback viscosity. The results were presented as mean ± standard deviations within the same column for each sample, with the application of different lowercases to indicate significant differences (*p* < 0.05).

**Table 2:** Gelatinization temperatures and enthalpy of WS–BSP mixtures.

BSP Conc.	To (°C)	Tp (°C)	Tc (°C)	ΔH (J/g)
0%	55.69 ± 0.17 a	61.13 ± 0.21 a	65.69 ± 0.33 a	1.566 ± 0.025 a
0.05%	55.70 ± 0.07 a	61.17 ± 0.09 a	65.65 ± 0.24 a	1.557 ± 0.012 a
0.1%	55.77 ± 0.05 a	61.13 ± 0.10 a	65.54 ± 0.19 a	1.533 ± 0.024 a
0.2%	55.89 ± 0.12 ab	61.29 ± 0.13 a	65.81 ± 0.20 a	1.510 ± 0.073 a
0.3%	55.98 ± 0.10 b	61.29 ± 0.07 a	65.57 ± 0.12 a	1.500 ± 0.009 a

The results are presented as mean ± standard deviations within the same column for each sample, with the application of different lowercases to indicate significant differences (*p* < 0.05).

**Table 3:** Steady flow parameters of WS–BSP gels.

BSP Conc.	Up				Down				Hysteresis Loop
	τ_0_ (Pa)	K (Pa·s^n^)	n (-)	R^2^	τ_0_ (Pa)	K (Pa·s^n^)	n (-)	R^2^	
0%	1.55 ± 0.17 a	0.97 ± 0.07 a	0.44 ± 0.05 a	0.995	1.07 ± 0.12 a	0.45 ± 0.02 a	0.55 ± 0.03 a	0.997	1342 ± 101 a
0.05%	2.94 ± 0.21 b	0.87 ± 0.09 a	0.49 ± 0.01 ab	0.995	1.48 ± 0.16 b	0.50 ± 0.08 ab	0.57 ± 0.06 a	0.997	2042 ± 127 b
0.1%	4.07 ± 0.29 c	1.38 ± 0.13 b	0.48 ± 0.04 ab	0.996	3.51 ± 0.23 c	0.57 ± 0.04 b	0.60 ± 0.02 a	0.990	2960 ± 204 d
0.2%	5.66 ± 0.13 d	1.69 ± 0.05 c	0.50 ± 0.07 ab	0.995	3.76 ± 0.27 cd	1.11 ± 0.06 d	0.56 ± 0.07 a	0.996	3113 ± 237 d
0.3%	6.26 ± 0.38 e	1.26 ± 0.07 b	0.57 ± 0.05 b	0.997	3.97 ± 0.11 d	1.00 ± 0.03 c	0.60 ± 0.03 a	0.997	2490 ± 173 c

The results are presented as mean ± standard deviations within the same column for each sample, with the application of different lowercases to indicate significant differences (*p* < 0.05).

**Table 4:** The mechanical properties of paper samples.

BSP Conc.	Tearing Strength (mN·m^2^/S)	Folding Endurance	Tensile Strength (kN/m)	Breaking Elongation (%)
0%	3.47 ± 0.30 a	1.89 ± 0.05 a	1.17 ± 0.08 a	0.92 ± 0.16 a
0.05%	3.63 ± 0.43 a	2.05 ± 0.04 ab	1.25 ± 0.11 ab	1.07 ± 0.07 ab
0.1%	4.25 ± 0.28 b	2.14 ± 0.14 bc	1.31 ± 0.05 ab	1.10 ± 0.11 ab
0.2%	4.69 ± 0.25 b	2.28 ± 0.08 c	1.32 ± 0.12 ab	1.13 ± 0.10 ab
0.3%	4.75 ± 0.26 b	2.47 ± 0.13 d	1.43 ± 0.13 b	1.17 ± 0.04 b

The results are presented as mean ± standard deviations within the same column for each sample, with the application of different lowercases to indicate significant differences (*p* < 0.05).

## Data Availability

Data may be shared upon request.
